# Assessing the efficacy of the ovicidal fungus *Mucor circinelloides* in reducing coccidia parasitism in peacocks

**DOI:** 10.1038/s41598-024-61816-7

**Published:** 2024-05-18

**Authors:** João Lozano, Cristina Almeida, Eduardo Vicente, Daniela Sebastião, Antonio Miguel Palomero, Cristiana Cazapal-Monteiro, María Sol Arias, Manuela Oliveira, Luís Madeira de Carvalho, Adolfo Paz-Silva

**Affiliations:** 1 CIISA – Centre for Interdisciplinary Research in Animal Health, Faculty of Veterinary Medicine, University of Lisbon, Avenida da Universidade Técnica, 1300-477 Lisbon, Portugal; 2 Associate Laboratory for Animal and Veterinary Sciences (AL4AnimalS), 1300-477 Lisbon, Portugal; 3 Exoclinic – Clínica Veterinária de Aves e Exóticos, Quinta de Santo António, 1495-049 Miraflores, Portugal; 4 EGEAC – Empresa de Gestão de Equipamentos e Animação Cultural, Castelo de São Jorge, Rua de Santa Cruz, 1100-129 Lisbon, Portugal; 5 Control of Parasites Research Group (COPAR, GI-2120), Department of Animal Pathology, Faculty of Veterinary, University of Santiago de Compostela, 27002 Lugo, Spain; 6 cE3c – Centre for Ecology, Evolution and Environmental Changes & CHANGE – Global Change and Sustainability Institute, Faculdade de Ciências, Universidade de Lisboa, 1749-016 Lisbon, Portugal

**Keywords:** Peacocks, Coccidia, Predatory fungi, *Mucor circinelloides*, Mini-FLOTAC, Portugal, Fungi, Parasitology

## Abstract

The biological control of gastrointestinal (GI) parasites using predatory fungi has been recently proposed as an accurate and sustainable approach in birds. The current study aimed to assess for the first time the efficacy of using the native ovicidal fungus *Mucor circinelloides* (FMV-FR1) in reducing coccidia parasitism in peacocks. For this purpose, an in vivo trial was designed in the resident peacock collection (*n* = 58 birds) of the São Jorge Castle, at Lisbon, Portugal. These animals presented an initial severe infection by coccidia of the genus *Eimeria* (20106 ± 8034 oocysts per gram of feces, OPG), and thus received commercial feed enriched with a *M. circinelloides* suspension (1.01 × 10^8^ spores/kg feed), thrice-weekly. Fresh feces were collected every 15 days to calculate the coccidia shedding, using the Mini-FLOTAC technique. The same bird flock served simultaneously as control (t0 days) and test groups (t15–t90 days). The average *Eimeria* sp. shedding in peacocks decreased up to 92% following fungal administrations, with significant reduction efficacies of 78% (*p* = 0.004) and 92% (*p* = 0.012) after 45 and 60 days, respectively. Results from this study suggest that the administration of *M. circinelloides* spores to birds is an accurate solution to reduce their coccidia parasitism.

## Introduction

Galliformes kept under free-range conditions in farms, zoos and private collections are highly prone to gastrointestinal (GI) parasitism caused by coccidia of the genera *Eimeria* and *Isospora*, and by nematodes such as *Capillaria* spp., *Ascaridia galli*, *Heterakis* spp., *Trichostrongylus tenuis* and *Strongyloides* spp.^1–6^ .

Coccidia, in particular, are responsible for severe health concerns in domestic and exotic birds, being of clinical (characterized by swelling of the intestinal wall and hemorrhages, leading to diarrhea and/or hemorrhagic feces, and even to death) or subclinical importance (associated with limited enteritis and loss of fluids, leading to poor absorption of nutrients), with poultry coccidia being the most studied^3,7^, resulting in average economic losses for the poultry industry of nearly 12 € billion annually worldwide^8,9^. Moreover, a total of nine *Eimeria* species have already been described in peacocks, namely *Eimeria arabica*, *E. kharjensis*, *E. mandali*, *E. mayurai*, *E. mutica*, *E. pavonina*, *E. pavonis*, *E. patnaiki* and *E. riyadhae*^6,10,11^, and despite not being so well studied, it is known that *E. mutica* and *E. kharjensis* develop their pathogenic activity on peacock’s ileum^12^.

Prevention and treatment of avian GI parasitic infections is still frequently performed exclusively with antiparasitic drugs, whose incorrect use often leads to low treatment efficacies, drug resistance, accumulation of drug residues on carcasses and contamination of soil and ground-waters^13–17^ .

Over the last 30 years, efforts have been made by the scientific community to develop new complementary approaches for the integrated control of GI parasitic infections in animals, namely the use of predatory fungi, also referred in the literature as “nematophagous”, “helmintophagous” or “parasiticide fungi”^18^. These saprophytic filamentous fungi are mostly found in agricultural soil and animal feces^19–24^ , and their main functional characteristic relies on the ability to capture and destroy environmental forms of GI parasites (larvae, eggs, and oocysts), by means of mechanical and enzymatic activities, and thus breaking the parasites’ life cycles on their exogenous stages. The most known predatory fungal taxa are *Duddingtonia flagrans* (Dudd.) R.C. Cooke (1969), *Arthrobotrys oligospora* Fresen., (1850), and *Monacrosporium thaumasium* (Drechsler) de Hoog & Oorschot (1985), which are able to destroy nematodes’ infective larvae (L3) (larvicidal fungi), whereas *Pochonia chlamydosporia* (Goddard) Zare & W. Gams (2001) and *Mucor circinelloides* Tiegh (1875) present ovicidal activity towards helminth eggs and coccidian oocysts (ovicidal/coccidicidal fungi)^18,25,26^.

Despite the majority of studies have addressed the use of these fungi to control ruminants^27–29^ and horses^30–32^ GI parasites, research on this topic has been recently extended to other animal species, namely dogs, birds, and captive wild animals^24,33–37^ . Regarding bird parasite control, it has been revealed that *P. chlamydosporia*, *D. flagrans*, *Arthrobotrys* spp. and *M. thaumasium* are promising candidates for the biological control of helminth infections, namely in chickens, laying hens and ostriches, and also confirmed chlamydospores’ tolerance to the avian GI biochemical environment^38–42^. Recently, Lozano et al.^24^ described for the first time the isolation of seven native *Mucor* spp. isolates (*M. circinelloides* and *M. lusitanicus*) from chicken and peacock fecal samples, and confirmed their coccidiostatic and coccidicidal activity towards *Eimeria* sp. oocysts, with the isolate *M. circinelloides* FR1 achieving the highest efficacy on destroying coccidia oocysts. However, field trials using predatory fungi to control GI parasitic infections in birds are still lacking.

The current research aimed to evaluate in vivo the efficacy of *M. circinelloides* (FMV-FR1) in reducing coccidia infections in an ornamental peacock collection.

## Results

**Figure 1:**
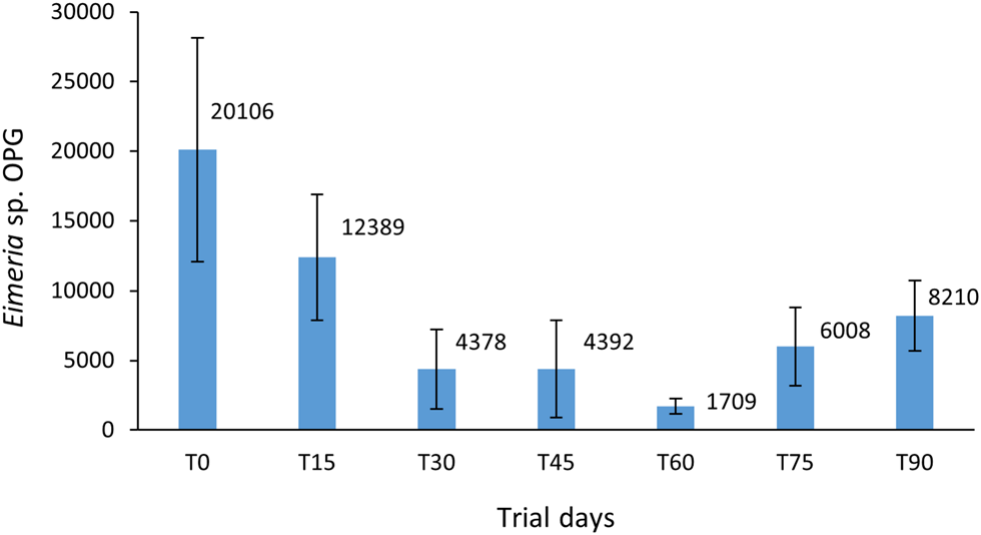
*Eimeria* sp. oocyst shedding dynamics during the trial in peacocks. Blue bars represent mean shedding values (± standard errors). Asterisks on days 45 and 60 mean significant differences in comparison with the control time point (*p* = 0.004 and *p* = 0.012, respectively).

Initial coprological results revealed that this peacock collection was severely infected with *Eimeria* sp. at the beginning of the trial (20107 ± 8034 oocysts per gram of feces, OPG). Following fungal administrations, it was possible to observe an overall decrease in oocysts’ shedding, with OPG reduction efficacies ranging between 59 to 92%, and with the decreased values at timepoints t45 and t60 being significant (*p* = 0.004 and *p* = 0.012, respectively) (Fig. 1). Furthermore, it was also observed that the average OPG did not reach the initial level during the entire assay.

**Figure 2:**
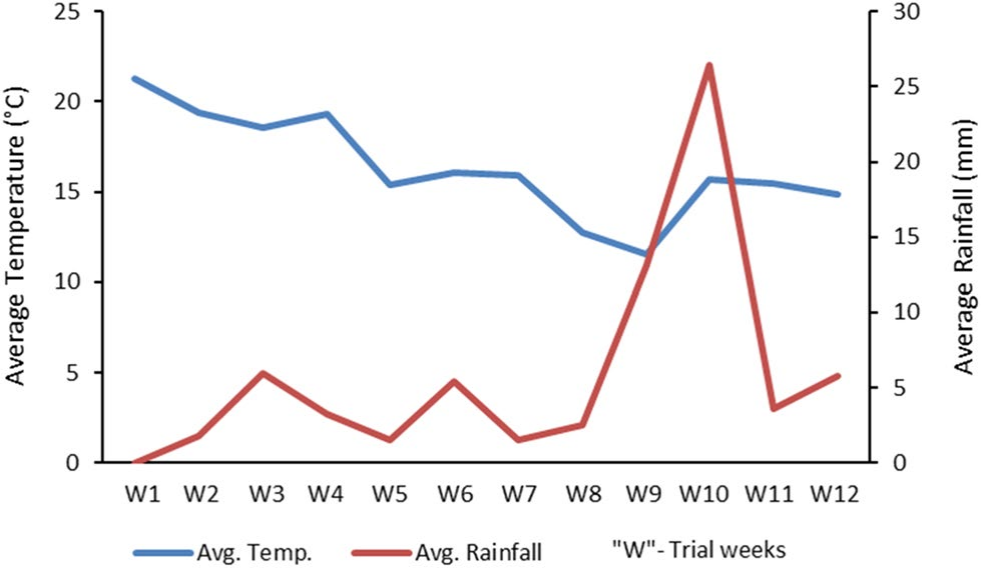
Average daily temperature (blue line, °C) and rainfall (red line, mm) recorded for the Lisbon Municipality, during the 12 weeks of the in vivo trial in peacocks (data retrieved from CLIMA.AML, https://www.clima.aml.pt).

Weather data tracked for the Lisbon municipality, in which the peacock collection was located, revealed an average temperature and rainfall of 16.4 °C (11.5–21.3 °C, min–max.) and 5.96 mm (0–26.4 mm, min–max), respectively, during the study period (Fig. 2). Also, a rainfall increase was observed in weeks 9 and 10, characterized by flood episodes in Lisbon downtown. Both temperature and rainfall did not significantly correlate with the coccidia shedding values (*p* = 0.12 and *p* = 0.48 for the correlations “*Eimeria* OPG—temperature”, and “*Eimeria* OPG—rainfall”, respectively).

Finally, no side effects were recorded after feeding peacocks with *M. circinelloides* spores, namely no changes on normal bird behavior, feed consumption, and feather appearance.

## Discussion

Predatory fungi are a promising solution for the integrated parasite management of animals kept in captivity in farms, zoos, and private collections, serving as a complement to the conventional antiparasitic drug treatments^25,36,43^. However, studies on this topic traditionally focus on horses and ruminants, and to our knowledge no large-scale field trials were previously performed aiming to assess the efficacy of predatory fungi in the reduction of avian GI parasitism.

Parasitological results from this study allowed to conclude that the parasiticide activity developed by *M. circinelloides* against *Eimeria* spp. oocysts reduced the environmental contamination by this parasite, with significant OPG reduction efficacies up to 92%, after 60 days of fungal feeding. These results demonstrated that the predacious activity developed by this type of fungi is a gradual process, as observed in other in vivo trials performed with *M. circinelloides* in dogs^26,36^, sheep^29^, baboons^36^ and wapitis^35^, which reported significant reductions in helminth egg shedding after a minimum of one month of routine fungal administrations.

The presence of parasitic forms (eggs, oocysts, and larvae) triggers predatory fungi hyphae germination and colonization of the fecal and surrounding soil’s microenvironments, with the final attachment to the oocyst/egg capsule prompting the first stage of ovicidal/coccidicidal activity^18,25,34^. This can be considered one of the most critical stages of the whole ovicidal process, together with the penetration of the parasite’s capsule, since fungi need to compete against other fecal and soil commensal microorganisms, attach on the parasite capsule and penetrate it through mechanical (appressorium and haustorium) and enzymatic activity (e.g., proteases, chitinases, collagenases and lipases)^25,44–47^ .

This trial was conducted mostly during the Autumn season, in which moderate average temperatures and rainfall were recorded in the Lisbon Municipality. Despite these weather variables being favorable for coccidia oocysts to reach the infective stage, which can even be accomplished at temperatures lower than 20 °C, and especially during Spring and Autumn seasons^48,49^, no significant correlations were obtained between weather and coccidia shedding. Thus, the possibility that meteorological conditions could contribute to differences observed in the parasitism results was discarded. Moreover, data collected regarding peacock samples suggest that this *M. circinelloides* isolate tolerated and maintained its predatory activity at overall temperatures and rainfall between 11.5–21.3 °C and 0–26.4 mm, respectively, being in accordance with previous in vitro and in vivo research using the strain *M. circinelloides* CECT 20824 for the biological control of strongyles, ascarids and trematodes^34,35,50^. It has also been demonstrated that *D. flagrans*, *P. chlamydosporia* and *Monacrosporium sinense* Xing Z. Liu & K.Q. Zhang (1994) maintain their germination capacity on different culture media even at temperatures lower than 20 °C^51,52^.

Despite the general OPG decreasing trend observed during the trial, following fungal spores’ administrations, a slight increase was recorded on the last two weeks, which coincided with the intense rainfall period recorded for the Lisbon district, as previously mentioned. Between t67–90 days of trial, and due to climate conditions, peacocks were not let free ranging in the monument’s outdoor area, and thus were sheltered in close contact with each other, leading to a higher exposition to feces contaminated with coccidia oocysts, and consequently stimulating re-infections. Avian *Eimeria* spp. have a very short life cycle, with a prepatent period of minimum four days^3,8^, and thus increasing bird stocking density can trigger re-infections and increase coccidia shedding^53^. These results also point out to the need for a constant monitorization of parasite shedding and clinical signs, during a parasite biocontrol program using predatory fungi, since treatments with antiparasitic drugs might be necessary to complement the action of these fungi.

This was also the first biological control trial to use the coprological technique Mini-FLOTAC for assessing the dynamics of coccidia shedding following the administration of predatory fungi to animals. Since 2014, there has been an increment in studies reporting the use of Mini-FLOTAC in routine diagnosis of GI parasitic infections in several animal species, being unanimously considered a good alternative to the traditional McMaster technique^54,55^ . Recent studies with Mini-FLOTAC in birds have revealed its usefulness for the diagnosis of coccidia and helminth infections, achieving sensitivities of up to 100%^56–60^. Since typical in vivo studies with predatory fungi always aim to compare control and test groups and obtain the respective oocyst/egg shedding or L3 reduction efficacies, the use of more sensitive coprological techniques like MF is of major importance, allowing to detect the real differences between groups and determine more statistically robust treatment efficacies.

Finally, one of the major concerns regarding the use of predatory fungi to control GI parasites is whether this kind of fungi can be harmful to birds, and during this trial no side effects were recorded following the administration of *M. circinelloides* spores to peacocks. These outcomes are in accordance with extensive research performed with this fungal species in horses, ruminants, and dogs^26,29,61,62^, in which authors confirmed the lack of pathogenicity of *M. circinelloides* spores to these animal species.

Results from this research offer the opportunity to develop further studies in the topic of parasite biocontrol. It would be interesting if further in vivo trials could include: (i) a wider period of research (e.g., 6–12 months of spores’ administrations and fecal collections), to evaluate the long-term efficacy of *M. circinelloides* in maintaining the coccidia fecal shedding at basal levels in birds, as extensively demonstrated for strongyles affecting domestic and exotic herbivores^27,30,35,63^; (ii) the separation of birds in test and control groups, and if possible according to their age (e.g., juveniles and adults), since younger birds are typically more prone to be infected by coccidia than adults^49,64^, and thus parasiticide fungi efficacy might differ between age groups; (iii) the collection of blood samples for haematological analysis to conclude more about the safety of this fungus, as previously performed in horses and ruminants fed with *M. circinelloides* and *D. flagrans* spores^29,62,65^; (iv) a previous characterization of the different *Eimeria* species residing in the GI tract of the respective birds, to check if the parasiticide efficacy of *M. circinelloides* would differ between *Eimeria* species.

To our best knowledge, this is the first report of an in vivo trial performed in exotic birds kept under real ornamental conditions, using a native ovicidal fungus previously isolated from birds, and aiming to evaluate its efficacy in reducing coccidia parasitism. This study revealed that feeding *M. circinelloides* to peacocks did not result in any side effects for birds, while achieving significant reduction efficacies of 78–92% in their coccidia parasitism. Overall results allow to propose *M. circinelloides* as a good fungal candidate for an accurate, safe, and sustainable parasite control program in birds.

## Methods

### Peacock collection

This trial was performed on the resident peacock collection (*Pavo cristatus*) of São Jorge Castle, a national monument located in Lisbon downtown, Portugal (38°42′50.241″ N 9°8′2.182″ W). The flock was composed by 58 birds (44 adults and 14 chicks), kept freely in an outdoor area of 4700 m^2^. According to previous research performed at the LPPD-CIISA-FMV, infections by *Eimeria* sp., *Capillaria* sp. and *Strongyloides pavonis* were identified in this peacock collection^59^.

These birds were normally fed twice per day with a formulation composed by corn, wheat, barley, sorghum, soyabean meal, sunflower seeds, calcium carbonate, monocalcium phosphate, soyabean oil, sodium chloride, molasses, and lard (nutritional composition: 15.8% Crude Protein, 4.25% Crude Fibre, 5% Ash, 3.45% Crude Fat, 0.8% Calcium, 0.43% Phosphorus, 0.09% Sodium, 0.75% Lysine and 0.32% Methionine), given ad libitum at two different locations.

In this bird collection, the assistant Veterinarians annually perform a single oral administration of Toltrazuril (25 mg/kg) to the juveniles (March–April), and the entire group receives febantel (15 mg/kg), pyrantel pamoate (5 mg/kg) and praziquantel (5 mg/kg), as a pre-mixture. However, no antiparasitic drug treatment was performed at least 6 months prior and during the trial.

### Preparation of the fungal suspension

A native *Mucor circinelloides* (FMV-FR1) ovicidal isolate, belonging to the predatory fungi collection of the Laboratory of Parasitology and Parasitic Diseases of the Faculty of Veterinary Medicine—University of Lisbon (LPPD-CIISA-FMV), whose parasiticide activity towards avian coccidia was previously confirmed^24^, was used in the current study. This isolate was stored in Wheat-Flour Agar (2%) medium, at room temperature, in a dark and dry environment, and cultured according to Arias et al.^66^. Briefly, a wheat broth was prepared, using 10 g of wheat grains per 1 L of distilled water. After autoclaving, 50 mL of broth were transferred to 20 plastic bottles, previously washed, and sterilized with UV-light. Wheat Agar cubes of 2.25 × 2.25 × 2.25 cm, containing mycelia from *M. circinelloides*, were cut and added to the broth in each bottle, which were then left at room temperature with a slope of 45° for one month. Finally, fungal suspensions of 10^6^ spores’ fold/mL were established, using a Neubauer chamber to count *Mucor* conidia and chlamydospores, and kept in a dry environment until its further use.

### Fungal administrations

**Figure 3:**
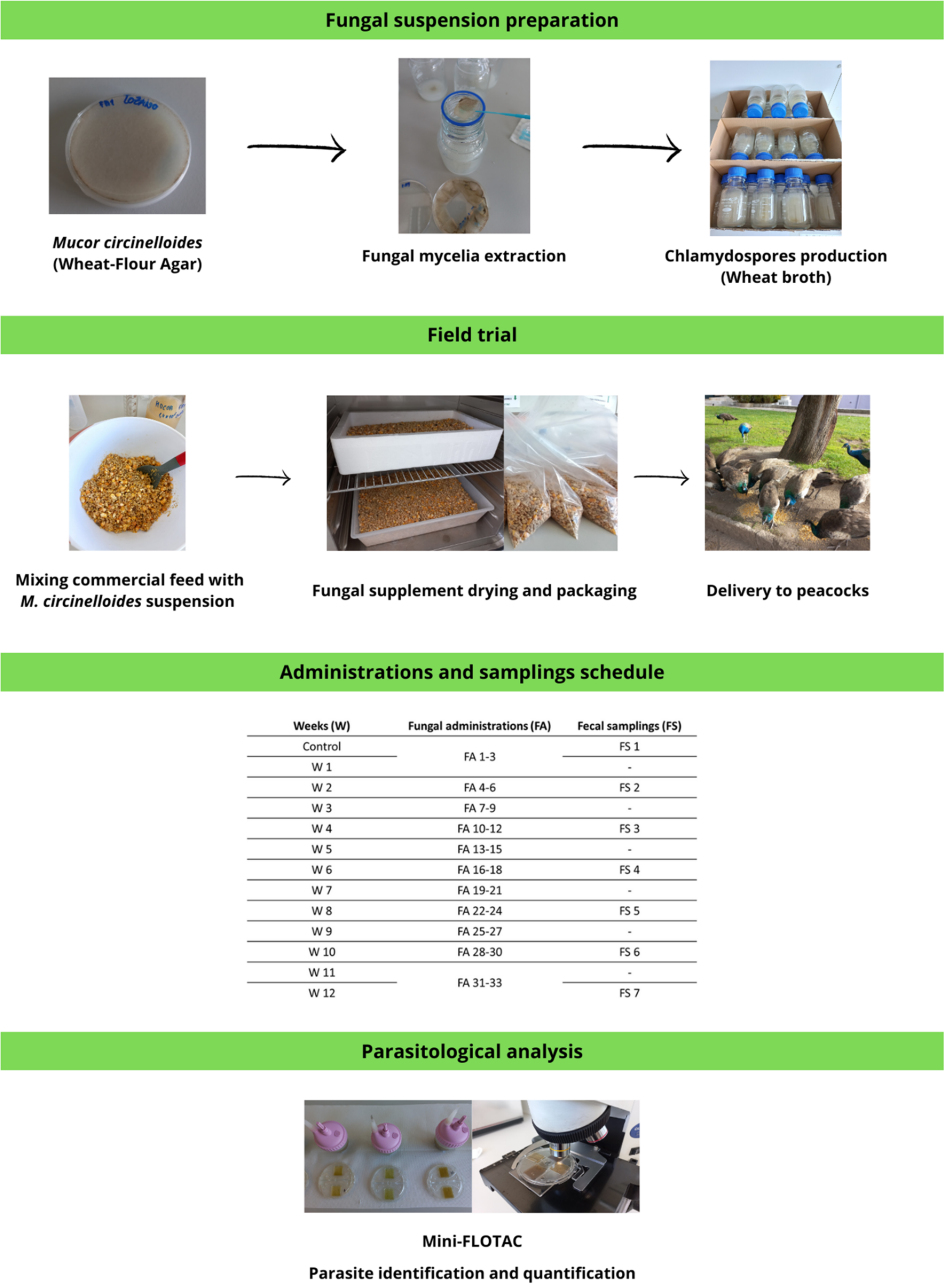
Experimental design of the in vivo trial performed in the selected peacock collection (figure created using Canva®; www.canva.com).

For this assay, bird feed doses containing *M. circinelloides* spores were previously prepared in the laboratory, following the protocol proposed by Voinot et al.^29^. For each dose, 600 g of peacock feed were mixed with 60 mL of the fungal suspension with 10^6^ spores/mL. The mixture was dried at 27 °C, for 30 min, using an incubator, and then packaged individually in sealed plastic bags, with peacocks receiving 1.01 × 10^8^ spores/kg of feed (Fig. 3).

The assay lasted between October—December 2022 and was based on the procedures described by Palomero et al.^35^ and Voinot et al.^29^. During this timeframe, a total of 33 fungal oral administrations were performed, thrice weekly. Since it was not possible to set two separate groups (test and control), the same flock served simultaneously as control (t0 days) and test group (t15–t90 days).

### Parasitological analysis

A total of 20 fresh fecal samples were collected from the environment, immediately after excretion, every 15 days, with exception for t0 days (*n* = 17 samples) and t30 days (*n* = 16 samples), and then individually packed in plastic bags and stored in a refrigerator (4 °C) at the LPPD-CIISA-FMV, for maximum one week.

All samples were processed using the coprological technique Mini-FLOTAC, to identify coccidia oocysts and helminth eggs, and calculate their fecal shedding (oocysts or eggs per gram of feces, OPG or EPG). For this purpose, the Mini-FLOTAC protocol followed the guidelines proposed by the manufacturer and referred in the literature for exotic animals^54,59,60^. Briefly, 2 g of feces were mixed with 38 mL of saturated sucrose solution (specific gravity 1.2), using the Fill-FLOTAC device; the resulting fecal suspension was transferred to the counting chamber, and left resting on the lab bench for 10 min, to allow parasitic forms to float and attach to the counting grids; then, the top disk was rotated clock-wise, and all coccidia oocysts and helminth eggs were identified and counted in an optical microscope (100x), using an analytic sensitivity of 10 OPG/EPG.

At the beginning of the trial, peacocks revealed very low burdens of *Capillaria* sp. (1.2 ± 0.81 EPG), which was the only helminth identified, and therefore only coccidia shedding was considered for further analysis.

The average coccidia OPG was calculated for each sampling timepoint, and treatment efficacy was determined based on the coccidia fecal oocyst count reduction (FOCR), using the following formula^30,31,35^:

$${\text{FOCR }}\left( \% \right) \, = \, [{1 }{-} \, \left( {{\text{OPG}}_{{\text{test day}}} /{\text{OPG}}_{{{\text{day }}0}} } \right]$$

### Side effects

Peacocks were regularly examined for any side effects resulting from fungal administrations, namely feed rejection, modifications on normal bird behavior, feathers appearance, skin lesions, and diarrhea or blood on droppings.

### Climate conditions

Weather data recorded in the Lisbon Municipality, for the 12 weeks of the trial, namely average temperature (°C) and rainfall (mm), were retrieved from the regional platform “CLIMA.AML: Rede de Monitorização e Alerta Meteorológico Metropolitano” (URL: https://www.clima.aml.pt).

### Statistical analysis

Calculations for mean values and standard errors were performed using the software IBM® SPSS® Statistics, version 27 for Windows (IBM Corporation, Armonk, NY, EUA), which was also used for all further statistical analysis. It was observed that the coccidia OPG data failed the Shapiro–Wilk normality test (*n* < 50 samples for every timepoint; *p* < 0.001). Thus, the coccidia OPG recorded in each test time point was compared with control using the Mann–Whitney test. Also, a correlation analysis was performed to assess a possible statistical association between coccidia OPG and the weather variables “average temperature” and “rainfall”, using the Spearman’s Test. A significance level of *p* < 0.05 was used for all statistical tests.

### Ethical approval

This study was approved by the Ethical Committee for Research and Teaching of the Faculty of Veterinary Medicine—University of Lisbon (CEIE 019/2022) and also received the written consent of the bird collection owner. Fungal administrations and fecal samplings were performed without any direct manipulation of the animals, and the study followed the normal daily procedures of the peacock collection.

All methods were carried out in accordance with relevant guidelines and regulations, as well as authors complied with the ARRIVE guidelines.

## Data Availability

The datasets generated during the current study can be made available by requesting it from the Corresponding Author.
